# High spatial resolution underwater data for mapping seagrass transplantation: A powerful tool for visualization and analysis

**DOI:** 10.1016/j.dib.2021.107735

**Published:** 2021-12-22

**Authors:** Daniele Ventura, Luca Castoro, Gianluca Mancini, Edoardo Casoli, Daniela Silvia Pace, Andrea Belluscio, Giandomenico Ardizzone

**Affiliations:** a Department of Environmental Biology, University of Rome ‘La Sapienza’ - V. le dell'Università 32, Rome 00185, Italy.; b Department of Engineering, Roma Tre University - Via della Vasca Navale, Roma 79-00146, Italy

**Keywords:** Structure from motion (SfM) photogrammetry, D scene rendering, Blender, Posidonia oceanica

## Abstract

These datasets derived from our mapping protocol are presented as a research article in the Journal of Environmental Management [1]. In particular, by using a Structure from Motion photogrammetric workflow we produced high spatial resolution 2D raster maps and 3D outputs such as dense points clouds and textured meshes of an underwater seagrass restoration site. In this area transplanted fragments of Posidonia oceanica were planted to restore this impacted site after the Costa Concordia shipwrecking which occurred on 13 January 2012 along the NE coast of Giglio Island (Tuscany, Italy). Photogrammetric outputs were used to render the underwater environment by using the open-source software Blender allowing a fine 3D modelling and immersive visualization of the mapped area. This data other than providing an exceptional tool for analysing the benthic habitats from a biological point of view, following over time the progress transplanting operations, might also provide a new way to visualize and share the perception of such underwater shallow environments to a large plethora of users, increasing the public awareness on restoration programmes and promoting new action aimed at restored underwater habitats restoration.

## Specifications Table

**Table:** 

Subject	Marine Biology Management, Monitoring, Policy and Law Nature and Landscape Conservation Education Management of Technology and Innovation Plant Science: General Bioengineering Planning and Development
Specific subject area	Underwater marine habitats mapping and 3D scene modelling
Type of data	Photos (RGB digital photos) Figures Maps 3D point cloud (.LAZ file (3D dense point cloud) 3D mesh models with texture (.OBJ) Video (Mp4 video)
How the data were acquired	The photographic data, which are the first step for data processing, were acquired with a low-cost action camera (GoPro Hero 9) capable of shooting high quality (20 Mpix images) photos with a specific time-lapse function. To perform the acquisition over a large area (3200 m^2^) the camera was mounted on a Diver Propulsion Vehicle (DPV) which allowed the SCUBA diver to map the entire area in only 1h and 10’. Consecutive (2 seconds interval) photos were acquired in raw format to allow further manual editing aimed at improving the result via colour grading and correction. Subsequently, each image was processed in a photogrammetric software (Agisoft Metashape) based on Structure from Motion (SfM) processing algorithms to generate 2D raster outputs (orthophoto mosaics and Digital Elevation Models, DEM),3D dense point clouds and 3D triangular mesh with texture. Finally, the open-source software Blender 2.93 was used to render the 3D scene to create a realistic view of the transplanted area by adding water effects and Posidonia leaves that otherwise cannot be well reproduced by only SfM data.
Data format	Raw and analysed
Description of data collection	The data were acquired by a SCUBA diver on 9 July 2020 during an underwater inspection of the transplanting site in the shipyard area along the NE coast of Giglio Island. The depth range investigated ranged from 6 up to 21 m depth.
Data source location	• Institution: University of Rome la Sapienza • City/Town/Region: Giglio Island • Country: Italy • Latitude and longitude: N 42.36490, E 10.92025
Data accessibility	Repository name: Mendeley Data. Data identification number: 10.17632/txjxfppp3w.1 Direct URL to data: https://data.mendeley.com/datasets/txjxfppp3w/draft?a=d1f6954b-bdc0-40fc-bc19-d76c4f11a9f5
Related research article	This data supports a research article that is accepted after revision in the Journal of Environmental Management (Paper id: YJEMA_114262)

## Value of the Data

• These data are useful because provides a new way to finely map benthic habitats where seagrass restoration programmes may occur. Detailed cartographic products are one of the main outputs required for long term monitoring actions which are often used in the assessment of marine impacted sites.
• These data can be used by a large number of users who are also non directly involved in marine restoration programmes. For instance, educational users can benefit from underwater visualization of benthic habitats as well as local stakeholders and managers.
• These data can provide baseline information for further studies aimed at investigating underwater mapping techniques as well as such information could help marine biologists in data sharing and visualization among the non-scientific community. Such detailed 3D reconstructions can then be used to show the status of underwater habitats, reducing the risk of incorrect organisms’ identifications and interobserver variability, through recording a permanent and reviewable record. In addition, fly-through visualizations can provide a safe and easier-to-access virtual environment also to non-specialized personnel that could be used to raise awareness on marine biota by providing to the younger public an interactive experience where they can “dive” through these sites, making them feel more involved and better aware of the need for further habitat exploration and conservation.

## 1 Data Description

 
*Data available on the online repository (folders’ names on Mendely are in bold)*
 *3D point cloud →* .Laz file: 3D dense point cloud generated after SfM processing in Agisoft Metashape.
 *3D textured mesh →* .Obj file: 3D mesh model with associated textures (.jpg)
 *Underwater RGB images→* Underwater images: Gopro Hero 9 action camera RGB images derived by underwater mapping carried out by SCUBA diver aided by DPV.
 *Video →* . mp4 video file of the reconstructed transplantation area rendered in Blender v. 2.93 with applied effects (*Posidonia* leaves and water).
 *Figures:* Main figures reported in the materials and methods section.

## 2 Experimental Design, Materials and Methods

**Fig. 1:**
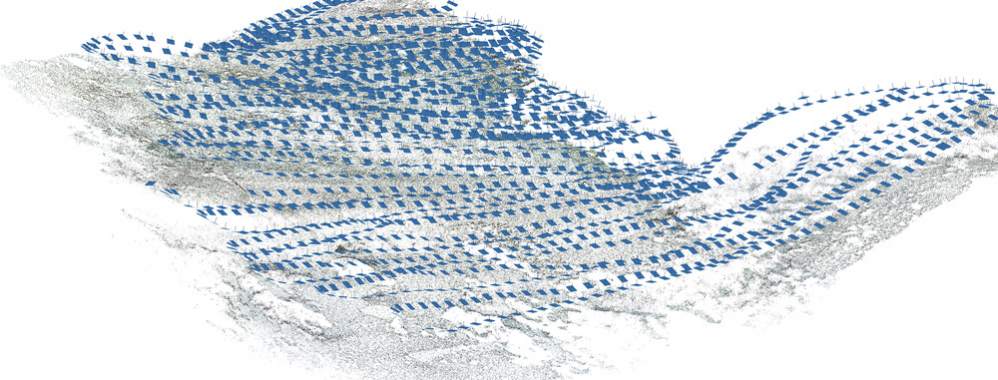
3D sparse point cloud of the transplantation area and camera locations (represented by blue frames) generated after photos alignment in Agisoft Metashape v.1.7.2.

**Fig. 2:**
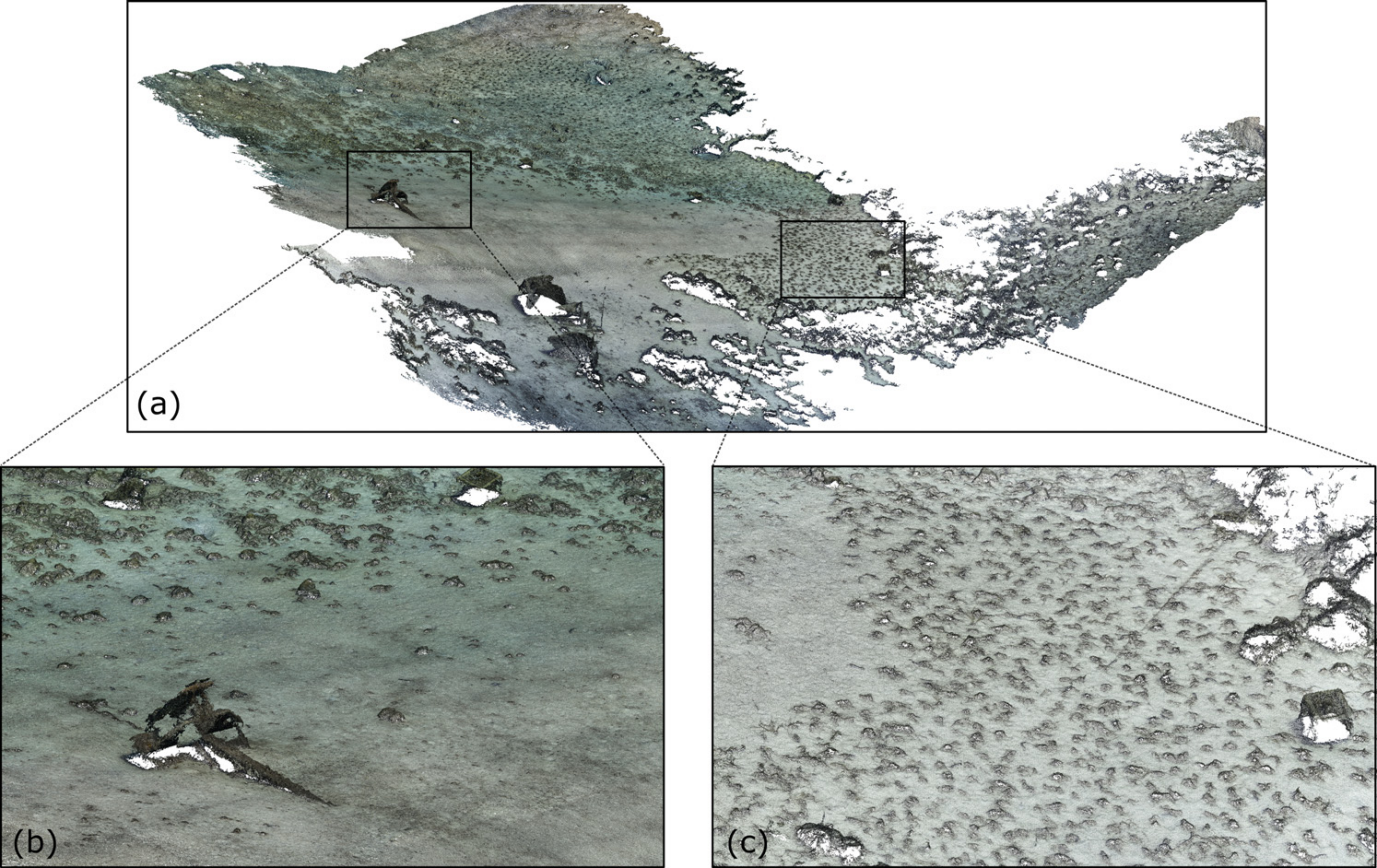
3D dense point cloud of the transplantation area generated by Multi-View Stereo (MVS) algorithm in Agisoft Metashape v.1.7.2. (a) General view of the whole area. (b) Detailed view of large anchor lying on the seabed. (c) Detailed view of the transplanted *Posidonia* fragments.

**Fig. 3:**
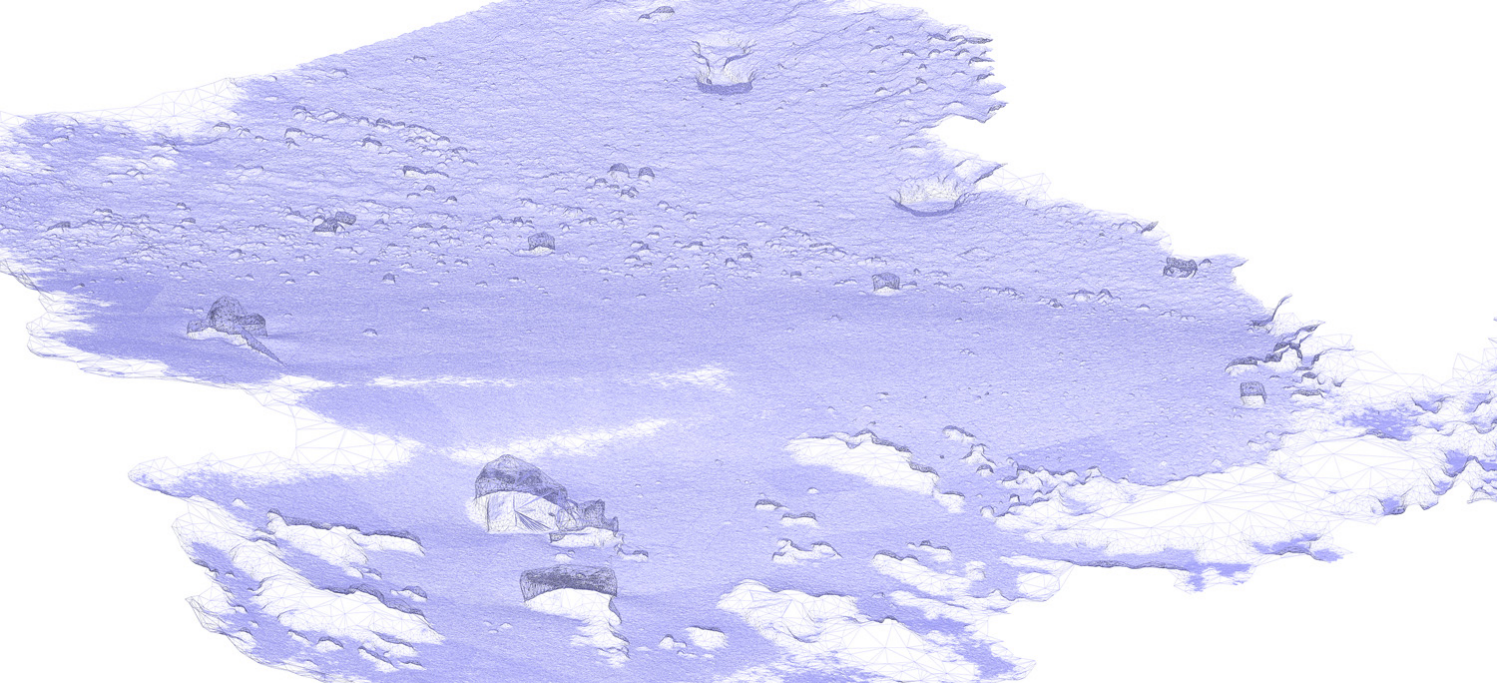
3D wireframe mesh model of the transplantation area.

**Fig. 4:**
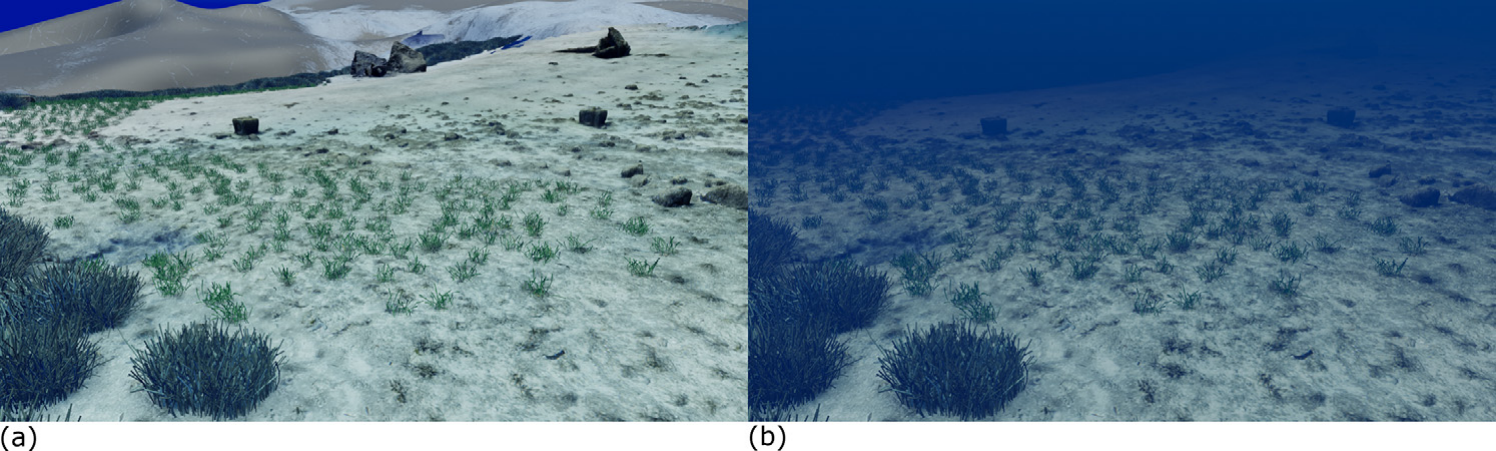
Video frames representing underwater scene reconstruction and rendering carried out in the open-source software Blender v. 2.93. *Posidonia oceanica* leaves were added by using the “hair” algorithm applied on the particle emitter associated with vertex groups that are manually highlighted on specific areas of the model by the “weight paint” tool. (a) Scene rendered without water effect. (b) Scene rendered with water effect.

**Fig. 5:**
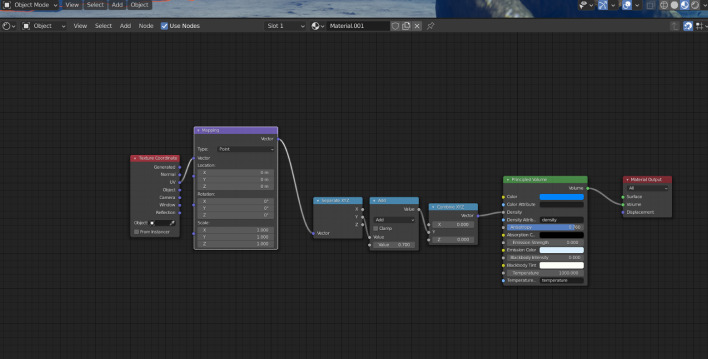
Shading panel in Blender v. 2.93 showing the customized parameters to generate the water effects as volumetric fog.

Before importing the imagery into Agisoft Metashape, the GoPro photographs were recorded with Protune mode activated to record a flat colour profile (raw) which is more suitable for colour enhanced t(white balance, colour contrast and saturation, exposure, Shadows/Highlights) which was subsequently carried out in Adobe Lightroom (v. 5.1). Three major steps were involved to process images in Metashape to reconstruct a 3D model of the seabed (Ventura et al., In press). First, an alignment of the photographs was executed by using an approach similar to the Scale Invariant Feature Transform (SIFT) algorithms. During this step, image pixels that are stable under viewpoint and lighting variations, are used to detect features points across overlapping photos and, then, to compute camera interior and exterior orientation parameters [2,3]. Using this information as input, the locations and movement of those feature points were monitored throughout a sequence of multiple images with different angles to estimate camera location and to render a sparse point cloud [4,5] (Fig. 1). At this step, ground control targets (GCPs) whose coordinates were surveyed by high accuracy GPS, are identified within the surveyed scene and identified on each photo in which they are visible to geolocate and scale the photogrammetric products. During the second step, the software used a Multi-View Stereo (MVS) algorithm to create a 3D dense point cloud (Fig. 2). The dense point cloud generated with “medium” quality and “mild” depth-filtering settings, resulted in 160 million points (20,000 to 50,000 points/m^2^). Finally, the dense 3D point cloud was used to create a 3D triangular mesh (Fig. 3) of the scene geometry and a digital surface model (DSM). After mesh texturing executed in Metashape (Texture type: diffuse map; Mapping mode: adaptive orthophoto; Blending mode: mosaic; Texture size: 4096 × 4096 pix atlas), a 3D model was exported in OBJ format and then imported into the open-source software Blender 2.93 [6] for additional 3D modelling and effects rendering (Fig. 4). In this software the use of the “weight paint” tool allowed us to mark areas where natural Posidona patches and transplanted fragments resulted in a poor quality of the 3D mesh after SfM processing (due to movement of seagrass leaves which occurred also with very low currents and good sea conditions). The highlighted areas were then associated with a vertex group in which a particle emitter was used to simulate *Posidonia* leaves. The best results in creating a realistic leaf-like cluster were achieved using a hair particle emitter with 30000 instances, 0.08 m hair length, 2000 jittering amount, Newtonian physics type with 3 kg mass, diameter root 0.003 m and diameter tip set to 0.008 m. Note that these parameters are not directly linked to any biological characteristic of the seagrass because the native use of the algorithm imply the generation of hairs and furs. Finally, to render the water effect and realistic lighting we used volumetric fog (shading settings are provided in Fig. 5) and sunlight with a strength of 8000 Watt/m^2^.

## CRediT authorship contribution statement

**Daniele Ventura:** Conceptualization, Methodology, Software, Visualization, Formal analysis, Data curation, Writing – original draft. **Luca Castoro:** Conceptualization, Methodology, Software, Visualization, Formal analysis, Data curation, Writing – original draft, Writing – review & editing. **Gianluca Mancini:** Writing – review & editing. **Edoardo Casoli:** Writing – review & editing. **Daniela Silvia Pace:** Writing – review & editing. **Andrea Belluscio:** Supervision, Writing – review & editing. **Giandomenico Ardizzone:** Supervision, Writing – review & editing.

## Declaration of Competing Interest

The authors declare that they have no known competing financial interests or personal relationships that could have appeared to influence the work reported in this paper.
